# Bridging practices prior to brexucabtagene autoleucel for mantle cell lymphoma in the United Kingdom: An analysis of modality, response, toxicity and survival

**DOI:** 10.1111/bjh.70357

**Published:** 2026-03-08

**Authors:** Maeve A. O'Reilly, William Wilson, Bernard Maybury, Andrea Kuhnl, Claire Roddie, Ben Uttenthal, Rod Johnson, Rajesh Alajangi, Thomas Creasey, Ahmed Abdulgawad, Carlos Gonzalez Arias, Sunil Iyengar, Graeme Ferguson, Katerina Panopoulou, Alison Delaney, Angharad Pryce, Lourdes Rubio, Ceri Jones, Jonathan Lambert, Shweta Gupta, Amrith Mathew, Shenbagaram Kasivisvanathan, Olateni Awofisayo, Shreyas Hanmantgad, Graham P. Collins, Caroline Besley, Frances Seymour, Robin Sanderson, Sridhar Chaganti

**Affiliations:** ^1^ Department of Haematology University College London Hospital London UK; ^2^ Department of Haematology University College London Cancer Institute London UK; ^3^ Cancer Research UK and University College London Cancer Trials Centre London UK; ^4^ Department of Haematology University Hospital Birmingham Birmingham UK; ^5^ Department of Haematology Kings College Hospital London UK; ^6^ Department of Haematology Cambridge University Hospital Cambridge UK; ^7^ Department of Haematology Leeds Teaching Hospital Leeds UK; ^8^ Department of Haematology University Hospital Bristol Bristol UK; ^9^ Department of Haematology Newcastle upon Tyne Hospitals Newcastle UK; ^10^ Department of Haematology The Christie NHS Foundation Trust Manchester UK; ^11^ Department of Haematology The Royal Marsden Hospital London UK; ^12^ Department of Haematology Queen Elizabeth University Hospital Glasgow Scotland UK; ^13^ Department of Haematology Oxford University Hospital Oxford UK; ^14^ Department of Haematology Sheffield Teaching Hospital Sheffield UK; ^15^ Department of Haematology University Hospital Southampton Southampton UK; ^16^ Department of Haematology Manchester Royal Infirmary Manchester UK; ^17^ Department of Haematology Cardiff and Vale University Health Board Cardiff UK

**Keywords:** brexucabtagene autoleucel, bridging therapy, mantle cell lymphoma

## Abstract

Bridging therapy (BT) prior to brexucabtagene autoleucel (brexu‐cel) in mantle cell lymphoma (MCL) is supported by limited evidence. Here, we report BT modality and outcome in 176 patients at 15 centres in the United Kingdom. BT was delivered to 90% (158/176), the majority receiving standard chemotherapy +/− radiotherapy (53%) (SD chemo +/− RT) or targeted therapy (TT) alone (23%). Clinicians favoured SD chemo +/− RT in those with Eastern Cooperative Oncology Group Performance Status (ECOG PS) of 1, blastoid disease, bulk >5 cm and elevated lactate dehydrogenase. Overall response rate (ORR) was 46%. Higher ORR was observed with SD chemo +/− RT (58%), particularly R‐BAC (64%). Progressive disease despite BT was associated with a lower ORR to brexu‐cel (77% vs. 91%, *p* = 0.03) and a higher risk of ≥grade 3 ICANS (OR 3.43, 95% CI 1.44–8.10, *p* = 0.01). SD chemo +/− RT was associated with a higher incidence of ≥grade 3 neutropenia (Month 1), ≥grade 3 thrombocytopenia (Month 1, Month 3) and early non‐relapse mortality (<90 days, 13% vs. 0%) compared to TT alone. Neither BT modality nor response impacted progression‐free or overall survival post‐infusion. Review of haematopoietic reserve prior to the selection of BT regimen, rigorous management of delayed cytopenia post‐infusion and more effective and tolerable BT should be prioritised.

## INTRODUCTION

Brexucabtagene autoleucel (brexu‐cel) was granted conditional marketing authorisation by the European Medicines Agency (EMA) for mantle cell lymphoma (MCL) after ≥2 lines, including a Bruton's tyrosine kinase inhibitor (BTKi) (2020). ZUMA‐2 reported overall response (ORR) and complete response (CR) rates of 93% and 67%, respectively, with 37% in ongoing response at median follow‐up of 35.6 months.^1^, ^2^ In the real world, progressive disease (PD) is the primary reason for drop out between approval and infusion,^3^, ^4^, ^5^ compromising intention‐to‐treat (ITT) outcomes. Those who fail to reach chimeric antigen receptor T‐cell therapy (CAR T) infusion have a dismal overall survival (OS).^3^, ^4^

In ZUMA‐2, bridging therapy (BT) was limited to a BTKi and/or steroids^1^ and delivered to 37% of patients. In TRANSCEND NHL 001 (lisocabtagene maraleucel, liso‐cel), BT was delivered to 66% of patients, the majority receiving systemic therapy (71%) or combined systemic and radiotherapy (RT) (24%).^6^ In contrast, 68%–90% of real‐world patients receive BT prior to brexu‐cel.^3^, ^4^, ^7^, ^8^, ^9^ BT strategies are at the discretion of the treating physician, tailored to individual circumstances and supported by limited evidence. An understanding of which patients may benefit and which strategies confer superior outcomes would be valuable.

Akin to large B‐cell lymphoma (LBCL) where BT has been associated with adverse prognostic risk,^10^, ^11^ BT in MCL has been associated with higher risk features such as high Ki‐67, blastoid/pleomorphic variants and *TP53* aberrations.^8^ BT has also been associated with a higher incidence of ≥grade 3 ICANS^8^ and inferior expansion,^9^ potentially reflective of use in poorer risk candidates. Superior UK outcomes have been noted in LBCL patients achieving a CR or partial response (PR) to BT^12^ with a 42% reduction in PD and death post‐infusion. However, there are conflicting reports on the impact of BT in MCL.^3^, ^4^, ^8^

With an ORR to BT ranging from 22% to 41%^3^, ^7^, ^8^ and compromised ITT outcomes, key components of the success of CAR T in MCL include (a) identification of the most effective BT strategies and patients most likely to benefit (b) an understanding of the impact of BT on outcome and (c) access to more effective BT. Here, we report BT outcomes for 176 patients harvested for third‐line brexu‐cel in the United Kingdom.

## METHODS

### Patients

Consecutive patients approved by the National CAR T Clinical Panel (NCCP) (or Scottish equivalent) from 15 UK centres (Table S1) between February 2021 and July 2024 were included (ITT cohort). Data were collected retrospectively (REC reference: 24/EM/0221, IRAS project ID: 336254). Treatment eligibility has been described.^3^

### Definitions

BT included lymphoma‐directed therapy between harvest and lymphodepletion with the intention of stabilising disease. Use and choice of BT was at the discretion of the treating physician. BT was categorised into (a) no bridging or steroids only, (b) targeted therapy (TT) (BTKi, BCL2 inhibitor), (c) RT +/− TT, (d) rituximab +/− low‐dose chemotherapy and (e) standard chemotherapy +/− RT (SD chemo +/− RT). Low‐dose chemo included gentle oral regimens, delivered in an outpatient setting. Standard chemo included intravenous (IV) treatment, often associated with neutropenia and/or hospital admission. Patients who received any SD chemo +/− RT after harvest were included the SD chemo +/‐ RT group regardless of sequencing. Fludarabine and cyclophosphamide lymphodepletion (LD) was administered as per manufacturer's instructions. American Society for Transplantation and Cellular Therapy consensus guidelines^13^ graded cytokine release syndrome (CRS) and immune effector cell‐associated neurotoxicity syndrome (ICANS). Severe and life‐threatening infections were defined as requiring IV therapy or organ support/with symptoms of haemodynamic instability respectively.^14^, ^15^, ^16^ Organ function requirements, toxicity management and response assessments (Lugano 2014^17^) were determined locally.

### Statistical analyses

Associations between pretreatment factors and choice of BT were assessed using Fisher's exact and Wilcoxon rank‐sum tests for categorical and continuous variables respectively. Using univariate and multivariable logistic regression, associations were further examined for (a) SD chemo +/− RT and (b) TT alone. Response rates to BT and toxicity by type and response were compared using logistic regression. Kaplan–Meier and Cox regression assessed PFS and OS. Non‐relapse mortality (NRM) was analysed using Fine and Gray (relapse a competing risk).

## RESULTS

### Patient characteristics

**FIGURE 1:**
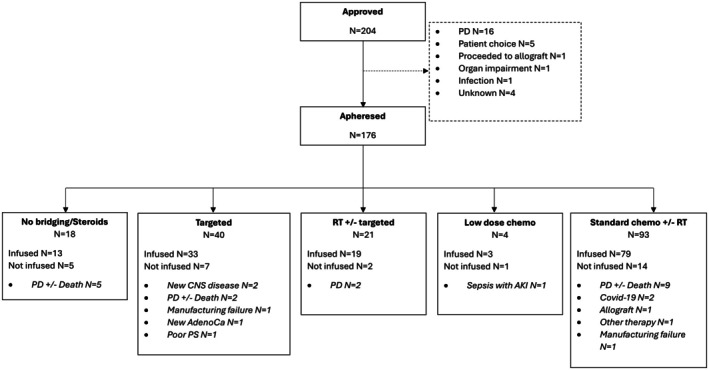
Intention‐to‐treat outcomes by type of bridging therapy.

One hundred and seventy‐six patients were harvested for brexu‐cel and BT was delivered to 90% (*n* = 158) (Figure 1, Table 1). 53% and 23% received a standard chemotherapy regimen and TT alone respectively. >1 chemotherapy regimen and/or BT modality were administered to 10% with the remaining 90% receiving one cycle and/or modality. R‐Bendamustine‐containing regimens were the most common (70/176, 40%) (Table S2).

**TABLE 1:** Patient demographics by modality of BT.

Characteristics	Bridging therapy					p value^a^					
	No bridging/steroids only, *N* = 18	Targeted, *N* = 40	RT +/− targeted, *N* = 21	Low‐dose chemo, *N* = 4	SD chemo +/− RT, *N* = 93	None vs. targeted	None vs. RT +/− targeted	None vs. SD chemo +/− RT	Targeted vs. RT +/− targeted	Targeted vs. SD chemo +/− RT	RT +/− targeted vs. SD chemo +/− RT
Age, median (range)	66 (56–74)	67 (46–78)	69 (49–77)	69.5 (58–74)	68 (41–81)	0.980	0.413	0.334	0.416	0.242	0.843
Sex											
Female	3 (17%)	10 (25%)	7 (33%)	1 (25%)	19 (20%)	0.735	0.290	1	0.555	0.648	0.250
Male	15 (83%)	30 (75%)	14 (67%)	3 (75%)	74 (80%)						
ECOG at submission											
0	8 (44%)	22 (55%)	9 (43%)	1 (25%)	25 (27%)	0.573	1	0.169	0.426	0.003	0.193
1	10 (56%)	18 (45%)	12 (57%)	3 (75%)	66 (73%)						
Unknown	0	0	0	0	2						
sMIPI at submission											
Low	3 (23%)	12 (32%)	4 (21%)	0	13 (16%)	0.783	0.658	0.849	0.605	0.130	0.383
Intermediate	4 (31%)	13 (34%)	9 (47%)	3 (75%)	27 (34%)						
High	6 (46%)	13 (34%)	6 (32%)	1 (25%)	40 (50%)						
Unknown	5	2	2	0	13						
HCT‐CI at submission											
0	6 (43%)	19 (51%)	7 (44%)	4 (100%)	46 (52%)	0.700	1	0.739	0.922	1	0.768
1 or 2	6 (43%)	11 (30%)	6 (38%)	0	27 (30%)						
≥3	2 (14%)	7 (19%)	3 (19%)	0	16 (18%)						
Unknown	4	3	5	0	4						
Ki‐67 at submission											
<30%	1 (10%)	5 (25%)	4 (24%)	0	9 (17%)	0.700	1	0.739	0.922	1	0.768
≥30%	9 (90%)	15 (75%)	13 (76%)	2 (100%)	45 (83%)						
Unknown	8	20	4	2	39						
Subtype at submission											
Blastoid	2 (15%)	4 (17%)	9 (50%)	0	35 (49%)	0.441	0.004	0.058	0.001	0.013	0.147
Classical	11 (85%)	15 (65%)	5 (28%)	2 (67%)	27 (38%)						
Leukaemic non‐nodal	0	2 (9%)	0	0	4 (6%)						
Pleomorphic	0	2 (9%)	4 (22%)	1 (33%)	6 (8%)						
Unknown	5	17	3	1	21						
*TP53* mutation											
No *TP53* mutation	6 (67%)	7 (35%)	9 (75%)	1 (100%)	21 (60%)	0.226	1	1	0.066	0.097	0.492
*TP53* mutation	3 (33%)	13 (65%)	3 (25%)	0	14 (40%)						
Unknown	9	20	9	3	58						
Stage at submission											
I–II	1 (6%)	4 (11%)	4 (20%)	1 (25%)	6 (7%)	0.685	0.055	0.208	0.198	0.435	0.107
III	0	3 (8%)	4 (20%)	0	14 (15%)						
IV	16 (94%)	31 (82%)	12 (60%)	3 (75%)	71 (78%)						
Unknown	1	2	1	0	2						
History of CNS disease											
No	18 (100%)	40 (100%)	21 (100%)	4 (100%)	87 (95%)	N/A	N/A	0.589	N/A	0.322	0.582
Yes	0	0	0	0	5 (5%)						
Unknown	0	0	0	0	1						
Bulk (>5 cm) at submission											
No	15 (88%)	30 (77%)	11 (58%)	4 (100%)	52 (57%)	0.473	0.065	0.015	0.218	0.047	1
Yes	2 (12%)	9 (23%)	8 (42%)	0	39 (43%)						
Unknown	1	1	2	0	2						
LDH at submission, median (range)	238.5 (161–694)	227 (105–2233)	242 (149–393)	207.5 (150–251)	262.5 (120–3313)	0.293	0.939	0.403	0.326	0.012	0.398
LDH at submission											
Normal	9 (50%)	24 (60%)	7 (39%)	2 (50%)	36 (40%)	0.571	0.738	0.442	0.163	0.037	1
Elevated	9 (50%)	16 (40%)	11 (61%)	2 (50%)	55 (60%)						
Unknown	0	0	3	0	2						
EN sites at submission											
0	1 (6%)	10 (26%)	8 (42%)	1 (25%)	30 (33%)	0.156	0.060	0.070	0.499	0.871	0.799
1	12 (71%)	15 (39%)	8 (42%)	2 (50%)	34 (37%)						
2	3 (18%)	8 (21%)	2 (11%)	1 (25%)	19 (21%)						
3	1 (6%)	5 (13%)	1 (5%)	0	8 (9%)						
4	0	0	0	0	1 (1%)						
Unknown	1	2	2	0	1						
Prior lines, median (range)	2.5 (2–6)	2 (2–7)	2 (2–5)	3 (2–4)	2 (2–5)	0.121	0.305	0.146	0.684	0.670	0.901
Time to 1st line progression (POD24)											
>24 months	10 (59%)	12 (32%)	5 (28%)	2 (50%)	38 (42%)	0.081	0.092	0.288	1	0.326	0.301
≤24 months	7 (41%)	25 (68%)	13 (72%)	2 (50%)	52 (58%)						
Unknown	1	3	3	0	3						
Primary refractory (to all lines)											
No	17 (94%)	33 (85%)	17 (85%)	4 (100%)	87 (94%)	0.413	0.606	1	1	0.180	0.196
Yes	1 (6%)	6 (15%)	3 (15%)	0	6 (6%)						
Unknown	0	1	1	0	0						
Ibrutinib refractory											
No	14 (82%)	26 (65%)	14 (70%)	3 (75%)	63 (69%)	0.224	0.462	0.385	0.777	0.686	1
Yes	3 (18%)	14 (35%)	6 (30%)	1 (25%)	28 (31%)						
Unknown	1	0	1	0	2						
Previous ASCT											
No	8 (44%)	26 (65%)	15 (75%)	2 (50%)	63 (68%)	0.162	0.096	0.067	0.560	0.841	0.603
Yes	10 (56%)	14 (35%)	5 (25%)	2 (50%)	30 (32%)						
Unknown	0	0	1	0	0						
Previous Allo‐SCT											
No	15 (83%)	34 (85%)	18 (86%)	3 (75%)	87 (94%)	0.162	0.096	0.067	0.560	0.841	0.603
Yes	3 (17%)	6 (15%)	3 (14%)	1 (25%)	6 (6%)						

Abbreviations: Allo‐SCT, allogeneic stem cell transplant; ASCT, autologous stem cell transplant; CNS, central nervous system; ECOG PS, Eastern Cooperative Oncology Group performance status; EN, extra‐nodal; HCT‐CI, Haematopoietic Cell Transplantation‐specific Comorbidity Index; LDH, lactate dehydrogenase; SD, Standard; sMIPI, simplified MCL international prognostic index.

^a^ Calculated using Fisher's exact test and Wilcoxon rank‐sum test.

Compared to TT alone, patients who received SD chemo +/− RT were more likely to have ECOG PS of 1 (73% vs. 45%, *p* = 0.003), blastoid MCL (49% vs. 17%, *p* = 0.01), bulk >5 cm (43% vs. 23%, *p* = 0.05) and an elevated lactate dehydrogenase (LDH) (60% vs. 40%, *p* = 0.04) (Table 1). In multivariable analysis (MVA), only ECOG PS 1 remained significantly associated with choice of SD chemo +/− RT over TT alone (OR = 2.96, 95% CI 1.06–8.22, *p* = 0.04) (Table S3).

### BT response and CAR T infusion rates

BT response assessment was available in 97% (*n* = 154). The ORR was 46% (71/154) with a CR rate of 12% (19/154). ORR was highest among those receiving SD chemo +/− RT (58%) compared to other modalities (OR = 3.24, 95% CI 1.64–6.40, *p* = 0.001) and lowest in TT alone (23%) (OR = 0.25, 95% CI 0.11–0.59, *p* = 0.001). Adjustment in MVA for ECOG PS, LDH, extra‐nodal (EN) sites and bulk did not alter these findings (Table 2) with patients receiving SD chemo +/− RT almost four times more likely to respond [OR = 3.79 (1.76–8.16), *p* = 0.001].

**TABLE 2:** Response to BT by modality.

Response	Targeted, *N* = 40	RT +/− targeted, *N* = 21	Low‐dose chemo, *N* = 4	Standard chemo +/− RT, *N* = 93
CR	3 (8%)	1 (5%)	0	15 (16%)
PR	6 (15%)	8 (38%)	1 (25%)	37 (40%)
SD	13 (33%)	4 (19%)	1 (25%)	14 (15%)
PD	17 (44%)	8 (38%)	2 (50%)	24 (26%)
Unknown (excluded)	1	0	0	3
ORR (95% CI)	23% (11–39)	43% (22–66)	25% (1–81)	58% (47–68)
Unadjusted	0.25 (0.11–0.59)	0.86 (0.34–2.17)	0.38 (0.04–3.75)	3.24 (1.64–6.40)
OR^a^ (95% CI), *p*‐value	*p* = 0.001	*p* = 0.7	*p* = 0.4	*p* = 0.001
Adjusted^b^ OR^a^ (95% CI), *p*‐value	0.26 (0.11–0.65) *p* = 0.004	0.62 (0.21–1.81) *p* = 0.4	N/A	3.79 (1.76–8.16), *p* = 0.001

Abbreviations: BT, Bridging therapy; CI, confidence interval; CR, complete response; ECOG PS, Eastern Cooperative Oncology Group performance status; LDH, lactate dehydrogenase; OR, odds ratio; ORR, overall response rate; PD, progressive disease; PR, partial response; SD, Standard.

^a^ Odds ratios calculated using logistic regression comparing response (CR or PR) in each modality to all other modalities combined.

^b^ Adjusted for ECOG PS, elevated LDH, ≥3 EN sites and bulk >5 cm (at submission).

When compared with other BT modalities, the use of rituximab, bendamustine and cytarabine (R‐BAC) chemotherapy^18^ alone was associated with a significantly higher ORR (64%, CR 16%). Those receiving R‐BAC were three times more likely to respond to BT (relative to other BT options) (OR = 3.06, 95% CI 1.54–6.08, *p* = 0.001). When compared with other SD chemo (excluding concomitant or sequential RT), the superior efficacy of R‐BAC trended towards significance (OR = 2.33, 95% CI 0.92–5.90, *p* = 0.07). Although the ORR with TT alone was only 23%, more favourable ORRs were noted in those who switched TT after approval (*n* = 13) with an ORR of 58% (7/12 with response data). Other TT included venetoclax (*n* = 9), venetoclax + ibrutinib (*n* = 2) and pirtobrutinib (*n* = 2).

No specific disease features such as BTKi refractoriness, subtype, progression of disease within 24 months of front‐line therapy (POD24), bulk, *TP53* mutation status or LDH were associated with response to BT. The only significant predictor of response was use of SD chemo +/− RT, with the strongest signal for R‐BAC chemotherapy.

Of 176 apheresed patients, 147 (84%) received their infusion. Infusion rates did not differ by BT modality (Fisher's exact *p* = 0.5) (Figure 1). Likewise, BT choice had no impact on time from harvest to cell infusion (Kruskal–Wallis *p* = 0.7) (Figure S1). Median time from harvest to infusion for all was 38 days (range 26–271).

### Best overall response to CAR T and survival by type and response to BT

**FIGURE 2:**
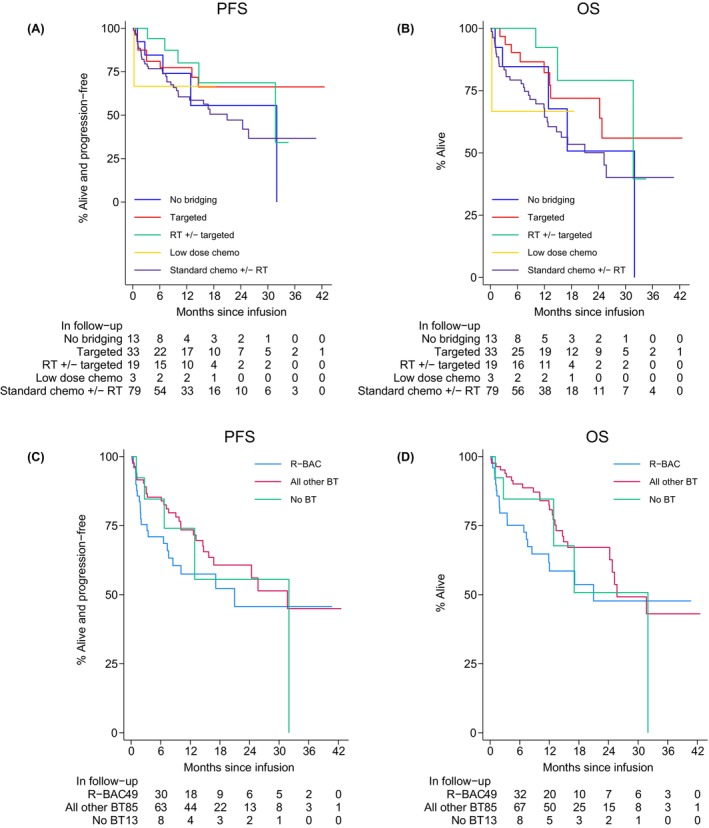
(A) Progression‐free survival (PFS) by type of BT; (B) overall survival (OS) by type of BT. (C) Progression‐free survival (PFS) post R‐BAC BT and (D) overall survival (OS) post R‐BAC BT. PFS (R‐BAC vs. all other BT): UVA: Hazard ratio (HR) = 1.47 (95% CI 0.84–2.58), *p* = 0.2; MVA: HR = 0.94 (0.51–1.71), *p* = 0.8. OS (R‐BAC vs. all other BT): UVA: HR = 1.60 (0.89–2.87), *p* = 0.1; MVA: HR = 1.01 (0.54–1.89), *p* > 0.9.

**FIGURE 3:**
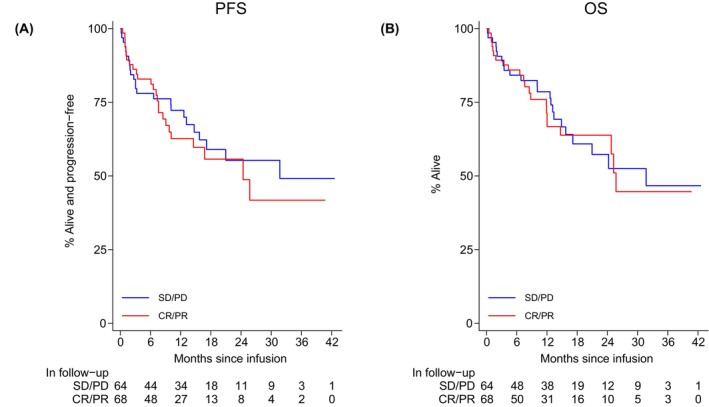
(A) Progression‐free survival* (PFS) by response to BT and (B) overall survival* (OS) by response to BT. *Includes those who received BT with available response data. PFS CR/PR versus SD/PD HR = 1.14 (95% CI 0.65–2.01), *p* = 0.6, OS CR/PR versus SD/PD HR = 1.07 (95% CI 0.59–1.92), *p* = 0.8.

The best ORR to CAR T was 87% (81% CR, 6% PR) with no demonstrable impact by BT modality. However, PD despite BT was associated with a lower ORR to CAR T compared with stable disease (SD), PR or CR to bridging (77% vs. 91%, *p* = 0.03) (Table S4). Twelve‐month PFS and OS rate for infused patients was 68% (95% CI 59–75) and 75% (95% CI 67–82) respectively. There were no significant differences between PFS and OS by type of BT (Figure 2A,B). Similarly, PFS and OS outcomes were unaffected by BT response (Figure 3A,B). Despite a significantly higher ORR to BT with SD chemo +/− RT compared to TT alone, no significant difference in survival post‐infusion was observed (PFS HR = 1.78, 95% CI 0.85–3.71, *p* = 0.1; OS HR = 1.86, 95% CI 0.89–3.89, *p* = 0.1) (Figure S2a,b). On MVA, factors associated with inferior PFS post‐infusion included history of CNS disease, POD24, elevated LDH and platelets <75 × 10^9^/L (Table S5).

No difference in ORR to CAR T was observed in recipients nor responders to R‐BAC alone (*n* = 58). PFS and OS post R‐BAC BT were not significant on univariate analysis (UVA) nor MVA when adjusted for factors which likely influenced BT choice at submission (ECOG PS, bulk and LDH) (Figure 2C,D).

### Toxicity by type and response to BT

Of 147 infused, 94% (*n* = 138) developed CRS with ≥grade 3 in 13% (*n* = 18, grade 5 *n* = 1). Factors associated with ≥grade 3 CRS on UVA included ECOG PS of 1 (17% vs. 5%, *p* = 0.04), ≥3 EN sites (42% vs. 10%, *p* = 0.01) and 2 (vs. 3) prior lines (17% vs. 4%, *p* = 0.03). Modality and response to BT had no impact on maximum grade (Figure S3a). However, those with PD despite BT (vs. SD/PR/CR) were more likely to require tocilizumab for CRS (30% vs. 9%, *p* = 0.04). ICANS occurred in 61% (*n* = 89) with ≥grade 3 in 24% (*n* = 35). While BT choice had no impact on maximum grade, patients with PD despite BT were over three times more likely to develop ≥grade 3 ICANS (OR 3.43 (95% CI 1.44–8.10), *p* = 0.01), occurring in 40% of those who progressed prior to infusion (Figure S3b). No other factors associated with ≥grade 3 ICANS were identified. BT choice and response had no impact on requirement for steroids or intensive care. 31% had a severe or life‐threatening infection within 30 days. Neither BT modality nor response impacted this risk (Table S6).

### ≥Grade 3 thrombocytopenia and neutropenia

Patients who received SD chemo +/− RT were more likely to experience ≥grade 3 thrombocytopenia at Month 1 (OR = 3.80, 95% CI 1.80–8.01, *p* < 0.001) and Month 3 (OR = 3.46, 95% CI 1.52–7.89, *p* = 0.003) versus other modalities combined. Those who received TT alone were less likely to have ≥grade 3 thrombocytopenia at Month 1 (OR = 0.17, 95% CI 0.07–0.40, *p* < 0.001) and Month 3 (OR = 0.25, 95% CI 0.08–0.80, *p* = 0.02) (Table S7a,b) versus other modalities combined.

Those who received SD chemo +/− RT were also more likely to experience ≥grade 3 neutropenia at Month 1 (OR = 2.22, 95% CI 1.09–4.55, *p* = 0.03) (vs. other modalities combined) but not at Month 3 (1.79, 95% CI 0.77–4.17, *p* = 0.2). Patients who received TT alone were less likely to experience ≥grade 3 neutropenia in Month 1 (OR = 0.41, 95% CI 0.18–0.93, *p* = 0.03) but no significant difference is seen at Month 3 (OR = 0.55, 95% CI 0.19–1.62, *p* = 0.3) (Table S7a,b). BT response had no impact on the incidence of ≥grade 3 neutropenia or thrombocytopenia.

### Non‐relapse mortality (NRM)

With median follow‐up from infusion of 16 months (interquartile range (IQR) 9.2–25.8), TT had a lower cumulative NRM risk relative to no bridging (subhazard ratio (SHR) = 0.07, 95% CI 0.01–0.51, *p* = 0.009), rituximab +/− low dose chemo (SHR = 0.07, 95% CI 0.01–0.98, *p* = 0.05) and SD chemo +/− RT (SHR = 0.11, 95% CI 0.01–0.82, *p* = 0.03) (Figure S4a, Table S8a). Notably, patients bridged with TT and SD chemo +/− RT were more likely to be ECOG PS 0 and 1 at submission respectively (Table 1). Adjusted for ECOG PS, the lower cumulative NRM risk of TT versus SD chemo +/− RT still trended towards significance [SHR 0.13 (95% CI 0.02–1.07), *p* = 0.06]. NRM risk with R‐BAC (vs. all other BT) did not reach significance (Figure S4b).

Patients receiving TT were also less likely to have an early NRM event (<90 days) (0%) relative to patients receiving more intensive regimens (13%) (Figure S4c, Table S8b). There was no difference in cumulative NRM (SHR = 1.10, 95% CI 0.48–2.50, *p* = 0.8) nor early NRM rates (10% [6/61] vs. 4% [4/63], *p* = 0.5) between bridging responders and non‐responders (Figure S5).

Other factors associated with early NRM included ECOG PS >1, elevated LDH, ≥grade 3 neutropenia at Month 1, severe or life‐threatening infection and ≥grade 3 ICANS (Table S9). Of note, 10/13 early NRM events were recipients of SD chemo +/− RT and the majority (8/10, 80%) had not recovered their neutrophils to >0.5 × 10^9^/L at death. Sepsis contributed to death in 63% (5/8) of cases (Table S10).

## DISCUSSION

Aside from symptom control and patient stabilisation, rationale for BT in MCL has been extrapolated from LBCL where markers of burden and inflammation (LDH, bulk, metabolic tumour volume) appear to correlate with inferior efficacy and/or toxicity outcomes.^12^, ^19^, ^20^, ^21^, ^22^, ^23^ Emerging data suggest that such variables may also compromise survival and/or safety in MCL.^3^, ^8^, ^9^, ^24^ However, the evidence underpinning BT, knowledge of the relative efficacy of modalities and impact on outcome is limited. Although the French (*n* = 152) reported a superior 12‐month OS for responders versus non‐responders to BT (79.9% vs. 58%),^4^ United States (*n* = 168) and United Kingdom (*n* = 83), authors reported no impact of BT or BT response on survival.^3^, ^8^

90% of UK harvested patients received BT. With this majority, it was not possible to determine which factors influenced the decision to administer BT (vs. watch and wait). BT was highly heterogeneous and likely guided by disease burden and pace, prior therapy/sensitivity, physician preference, patient fitness, haematopoietic reserve and access to novel therapies. Clinicians favoured SD chemo +/− RT in higher risk cases, with ECOG PS 1 the apparent main driver. The decision to deliver BT and/or more intensive BT in poorer risk candidates has been demonstrated in MCL^8^ and LBCL^11^, ^12^ and may confound assessment of outcomes. Therefore, comparisons of SD chemo +/− RT versus other modalities in our analysis should be interpreted with caution.

PD constituted the primary reason for drop out between apheresis and infusion (20/29, 69%). 84% of harvested patients received their infusion and BT modality had no clear impact on the risk of drop out nor time to infusion. Median time from harvest to infusion was 38 days (range 26–271), longer than ZUMA‐2 but comparable to US^8^ and European^7^ experiences. Reassuringly, there is no suggestion that adverse events secondary to BT increased morbidity or mortality pre‐LD. Nevertheless, BT response rates remained suboptimal at 46%. Those receiving SD chemo +/− RT were almost four times more likely to respond with the strongest signal for R‐BAC. The only clear predictor of response to BT was the use of SD chemo +/− RT. However, missing data on high‐risk features such as subtype, *TP53* mutation status and Ki‐67% may have limited this analysis. An alternative BTKi or a BCL‐2 inhibitor may deliver more favourable responses but small numbers precluded further analysis.

PD despite BT was associated with a lower ORR to CAR T (91% vs. 77%, *p* = 0.03). This lower ORR may be the consequence of higher burden at infusion or may reflect resistant disease biology. Regardless, our work supports the conclusion that neither use of nor response to BT impacts survival post brexu‐cel. This is despite our observation that disease burden variables (bulk >5 cm, LDH, ECOG PS > 1 and ≥ 3 EN sites) were associated with PFS. Notably, 61% of patients with a radiological CR after BT had a persistently elevated LDH, potentially accounting for the lack of a survival benefit in BT‐responders (Table S11). BT response was determined locally, primarily using PET‐CT. Bone marrows were not routinely performed to confirm CRs. Therefore, rates of CR (albeit low at 12%) may have been overestimated. However, response to BT was defined as achievement of CR/PR, and hence, it is unlikely that many BT responders were misclassified.

PD despite BT was associated with more frequent use of tocilizumab and higher risk of ≥grade 3 ICANS. High‐grade ICANS is of concern given the older median age in MCL, the higher incidence in elderly cohorts^25^, ^26^, ^27^, ^28^ and the NRM signal.^14^, ^29^ Risk of ≥grade 3 ICANS in BT non‐responders has been reported and validated externally in LBCL.^12^, ^30^ BT response had no impact on cumulative or early NRM risk. However, intensive regimens were associated with higher risk of early NRM (13%) versus TT (0%). The physician decision to administer an intensive regimen over TT is likely reflective of an adverse risk group. ECOG PS of 1 was significantly associated with choice of SD chemo +/− RT over TT. Although NRM risk for a more intensive regimen appeared to persist when adjusted for PS, there may be other factors contributing to higher NRM in such candidates. It should be acknowledged that intensive regimens were associated with a greater incidence of ≥grade 3 neutropenia at Month 1 and that this variable was also significant for early NRM. Vigilance and rigorous supportive care are required in the post‐infusion period to reduce the risk of early mortality. Severe immune effector cell‐associated haematotoxicity (ICAHT) is associated with higher incidence of severe infections, NRM and inferior survival outcomes.^31^ Rajeski et al. reported the increased incidence of severe ICAHT in MCL (brexu‐cel, 28%) relative to other B‐cell malignancies (LBCL 23%, myeloma 15%) and products. With adjusted analysis, marrow infiltration, ferritin and baseline cytopenia were associated with the severe phenotype of ICAHT.^31^ Clinicians should therefore be mindful that the selection of a BT regimen for MCL patients warrants careful consideration of haematopoietic reserve and inflammatory markers. If concern persists that a regimen may adversely affect the haematopoietic progenitor compartment but a response to BT is highly desirable, attenuated drug dosing can be considered. In the post‐infusion period, early GCSF, anti‐microbial prophylaxis and timely stem cell top up should be employed in select candidates. European Hematology Association/European Society for Blood and Marrow Transplantation consensus guidelines propose a treatment algorithm for ICAHT post CAR T.^32^

Our work is limited by retrospective data and local review of response. Data on hematotox scores and ICAHT grading were not collated. BT practices for brexu‐cel in the United States may differ, influenced by turnaround times, FDA label for brexu‐cel with no prerequisite for ≥2 lines and/or BTKi and approval of liso‐cel. A differential survival outcome between products and BT response has been noted in LBCL.^12^ Therefore, studies are needed to elucidate the impact of BT on safety and efficacy outcomes post liso‐cel.

With CAR T preferred for eligible MCL patients at ≥3rd line, new BT strategies are needed to minimise dropout. Pirtobrutinib, with an ORR of 57% in covalent BTKi‐exposed, may be a well‐tolerated and attractive option.^33^ Likewise, T‐cell engagers represent another emerging agent but are not yet accessible outside of clinical trials.^34^ The incorporation of BTKi's into front‐line therapy may modify future treatment pathways.^35^

In summary, standard chemotherapy regimens (particularly R‐BAC) are more likely to deliver a response prior to brexu‐cel. Those who responded to BT had a higher ORR to CAR T and lower risk of high‐grade ICANS, but no survival benefit was observed. Controlling disease with the least toxic regimen should be the goal of BT. Careful review of haematopoietic reserve prior to the selection of a BT regimen, rigorous supportive care for those with delayed cytopenia post‐infusion and more effective and tolerable BT strategies should be prioritised.

## AUTHOR CONTRIBUTIONS

Maeve A. O'Reilly: Conceptualizisation, supervision, data curation and writing the original draft. William Wilson: Conceptualizisation, formal analysis, data curation and writing the original draft. Bernard Maybury, Andrea Kuhnl, Claire Roddie, Ben Uttenthal, Rod Johnson, Rajesh Alajangi, Thomas Creasey, Ahmed Abdulgawad, Carlos Gonzalez Arias, Sunil Iyengar, Graeme Ferguson, Katerina Panopoulou, Alison Delaney, Angharad Pryce, Lourdes Rubio, Ceri Jones, Jonathan Lambert, Shweta Gupta, Amrith Mathew, Shenbagaram Kasivisvanathan, Olateni Awofisayo, Shreyas Hanmantgad, Graham P. Collins, Caroline Besley, Frances Seymour, Robin Sanderson, Sridhar Chaganti: Data curation, writing, review and editing.

## FUNDING INFORMATION

No funding received.

## CONFLICT OF INTEREST STATEMENT

MOR: honoraria from Kite Gilead, Novartis and Janssen. Advisory boards Kite Gilead and Autolus. Conference/travel support Kite Gilead. WW: No COI. BM: Conference support Kite Gilead. AK: Advisory boards and honoraria from Kite/Gilead, Novartis, Abbvie, Roche and BMS. CR: Advisory boards and speakers fees Novartis, Kite Gilead, BMS, Amgen, Autolus. BU: No COI. RJ: No COI. RA: No COI. TC: No COI. AA: No COI. CA: No COI. SI: conference support Beigene, BMS, Takeda. Speaker fees Kite Gilead, Takeda. Advisory boards Kite, MSD. GF: Course funding Abbvie. KP: No COI. AD: Advisory board and sponsorship for conference (Kite Gilead) and speaker fees (Roche and Abbvie). AP: No COI. LR: No COI. CJ: No COI. JL: No COI. SG: No COI. AM: No COI. SK: No COI. OA: No COI. SH: No COI. GC: Kite Gilead speaker fees, advisory board. CB: Honoraria Kite, Janssen, Novartis and Takeda. FS: No COI. RS: Kite Gilead speakers bureau, honoraria, conference travel, Novartis speakers bureau, honoraria, conference travel. SC: honoraria from Takeda, Kite/Gilead, Incyte, AbbVie, Pierre Fabre: F. Hoffmann‐La Roche Ltd., Atara Bio, Orion Pharma, Adicet Bio, Incyte, Novartis, Amgen, Sobi, Pierre Fabre, BMS‐Celgene and Miltenyi Biotec and meeting attendance support from Takeda, Kite‐Gilead, Abbvie and Pierre Fabre.

## ETHICS STATEMENT

REC reference: 24/EM/0221, IRAS project ID: 336254.

## PATIENT CONSENT

Consent acquired for use of data.

## Supporting information


Data S1.

## Data Availability

The data that support the findings of this study are available on request from the corresponding author. The data are not publicly available due to privacy or ethical restrictions.
