# Supplementary material for: Bridging practices prior to brexucabtagene autoleucel for mantle cell lymphoma in the United Kingdom: An analysis of modality, response, toxicity and survival
Source: Br J Haematol. 2026 Mar 8;208(4):1347–58. doi: 10.1111/bjh.70357 (PMC13071468; doi:10.1111/bjh.70357)

**Supplementary Tables and Figures**

**Supplementary Tables:**

| **CAR T centres** | |
| --- | --- |
| University College London Hospital, London | Newcastle upon Tyne Hospitals |
| University Hospital Birmingham | Royal Marsden London |
| Kings College Hospital, London | Sheffield Teaching Hospitals |
| Leeds Teaching Hospital | Manchester Royal Infirmary |
| Cambridge University Hospital | Cardiff and Vale University Health Board |
| University Hospital Bristol | University Hospital Southampton |
| The Christie NHS foundation Trust, Manchester | Oxford University Hospital |
| Queen Elizabeth University Hospital, Glasgow |  |

**Table S1: Participating CAR T Centres**

**Table S2: BT administered post apheresis**

| **Apheresed patients** | **N=176** | |
| --- | --- | --- |
|  |  | |
| **No bridging** | **17 (10%)** | |
|  |  | |
| **Steroids only** | **1 (1%)** | |
|  |  | |
| **Standard chemotherapy +/-RT** | **93 (53%)** | |
| R-BAC | 58 (33%) | |
| R-Bendamustine | 6 (3%) | |
| R-CHOP | 9 (5%) | |
| AraC+/- R | 7 (4%) | |
|  |  | |
| *Sequential BT containing any standard chemotherapy* | |  |
| PEP-C x 1, R-Bendamustine x 2 | 1 (1%) | |
| R-CHOP x 1, R-Bendamustine x 1 | 1 (1%) | |
| R-AraC, R- Vincristine/Dexamethasone | 1 (1%) | |
| R-Bendamustine x 1, Pirtobrutinib | 1 (1%) | |
| R-CVP x 1, R-BAC x 2, R-Lenalidomide x1 | 1 (1%) | |
| Ara-C + methylprednisolone x 1, Pirtobrutinib | 1 (1%) | |
|  |  | |
| *Sequential standard chemotherapy and RT* |  | |
| R-BAC + RT | 2 (1%) | |
| R-CHOP + RT | 2 (1%) | |
| AraC +/-R + RT | 3 (3%) | |
|  |  | |
| **Low-dose chemotherapy** | **4 (2%)** | |
| R | 3 2%) | |
| DECC | 1 (1%) | |
|  |  | |
| **Targeted therapy alone** | **40 (23%)** | |
| Ibrutinib | 26 (15%) | |
| Venetoclax | 9 (5%) | |
| Acalabrutinib | 1 (1%) | |
| Pirtobrutinib | 2 (2%) | |
| Venetoclax/ibrutinib | 1 (1%) | |
| Venetoclax/ibrutinib/rituximab | 1 (1%) | |
|  |  | |
| **RT +/- Targeted Therapy** | **21 (12%)** | |
| RT  RT + ibrutinib  RT + pirtobrutinib | 18 (10%)  2 (1%)  1 (1%) | |

R: Rituximab; R-BAC: rituximab, bendamustine, and cytarabine; RCHOP: rituximab, cyclophosphamide, doxorubicin, vincristine, prednisone; AraC: cytarabine; PEP-C: Prednisone, etoposide, procarbazine, cyclophosphamide; CVP; cyclophosphamide, vincristine, prednisolone; DECC: dexamethasone, etoposide, chlorambucil, lomustine; RT: radiotherapy

**Table S3. Univariate and multivariable logistic regression comparing standard chemo +/-RT to targeted BT**

|  | **UVA OR (95% CI), p-value** | **MVA OR (95% CI), p-value** |
| --- | --- | --- |
| Age (10-year increase) | 1.41 (0.89-2.24), p=0.1 | - |
| Sex (Male vs Female) | 1.43 (0.61-3.36), p=0.4 | - |
| ECOG (1 vs 0) | 3.37 (1.56-7.28), p=0.002 | 2.96 (1.06-8.22), p=0.04 |
| sMIPI at submission  Low  Intermediate  High | Reference  1.92 (0.69-5.35), p=0.2  2.64 (0.98-7.12), p=0.06 | - |
| HCT-CI at submission  0  1 or 2  ≥3 | Reference  0.93 (0.39-2.21), p=0.9  0.94 (0.33-2.66), p=0.9 | - |
| Ki67 at submission (≥30% vs <30%) | 1.67 (0.48-5.76), p=0.4 | - |
| Subtype at submission (Blastoid/pleomorphic vs Classical/leukaemic non-nodal) | 3.21 (1.19-8.70), p=0.02 | 2.45 (0.85-7.03), p=0.1 |
| TP53 mutation (Yes vs No) | 0.39 (0.12-1.23), p=0.1 | - |
| Stage at submission  I-II  III  IV | Reference  3.11 (0.53-18.38), p=0.2  1.48 (0.39-5.61), p=0.6 | - |
| History of CNS disease (Yes vs No) | 2.30 (0.26 (20.32), p=0.5 | - |
| Bulk (>5cm) at submission (Yes vs No) | 2.58 (1.10-6.05), p=0.03 | 1.79 (0.62-5.18), p=0.3 |
| LDH at submission (Elevated vs Normal) | 2.16 (1.02-4.57), p=0.05 | 1.27 (0.46-3.53), p=0.6 |
| EN sites at submission (≥3 vs <3) | 0.74 (0.23-2.36), p=0.6 | - |
| Prior lines (≥3 vs 2) | 1.30 (0.57-2.94), p=0.5 | - |
| POD24 (Yes vs No) | 0.63 (0.28-1.41), p=0.3 | - |
| Primary refractory (Yes vs No) | 0.33 (0.10-1.04), p=0.06 | - |
| Ibrutinib refractory (Yes vs No) | 0.77 (0.35-1.67), p=0.5 | - |
| Previous ASCT (Yes vs No) | 0.92 (0.42-2.00), p=0.8 | - |
| Previous Allo-SCT (Yes vs No) | 0.40 (0.12-1.33), p=0.1 | - |

ORs>1 indicate a higher chance of receiving standard chemotherapy +/-RT compared to targeted only BT.
MVA included all variables with p≤0.05 on UVA

**Table S4: Best ORR to CAR-T by BT modality and BT response**

| **BT modality** | **CR** | **PR** | **No response** |
| --- | --- | --- | --- |
| No BT/steroids only  (N=13) | 11/13 (85%) | 1/13 (8%) | 1/13 (8%) |
| Targeted (N=33) | 25/31 (81%) | 1/31 (3%) | 5/31 (16%) |
| RT +/- targeted  (N=19) | 17/18 (94%) | 1/18 (6%) | 0 |
| Low dose chemo  (N=3) | 2/3 (67%) | 0 | 1/3 (33%) |
| SD chemo +/-RT  (N=79) | 61/78 (78%) | 6/78 (8%) | 11/78 (14%) |
| **BT response** |  |  |  |
| CR  (N=19) | 16/17 (94%) | 0 | 1/17 (6%) |
| PR  (N=49) | 40/49 (82%) | 3/49 (6%) | 6/49 (12%) |
| SD  (N=30) | 23/28 (82%) | 4/28 (14%) | 1/28 (4%) |
| PD  (N=35) | 26/35 (74%) | 1/35 (3%) | 8/33 (23%) |

**Table S5: Univariate and Multi-variable analysis of factors associated with PFS post-infusion**

|  | **UVA HR (95% CI), p-value** | **MVA HR (95% CI), p-value** |
| --- | --- | --- |
| **At submission** |  | **-** |
| Age (10-year increase) | 1.20 (0.84-1.71), p=0.3 | - |
| Sex (Male vs Female) | 2.18 (1.03-4.63), p=0.04 | - |
| ECOG (1 vs 0) | 2.15 (1.18-3.92), p=0.01 | - |
| sMIPI  Low  Intermediate  High | Reference  0.88 (0.38-2.04), p=0.8  1.30 (0.61-2.75), p=0.5 | - |
| HCT-CI  0  1 or 2  ≥3 | Reference  1.18 (0.64-2.18), p=0.6  0.98 (0.43-2.27), p>0.9 | - |
| Ki67 (≥30% vs <30%) | 1.42 (0.54-3.73), p=0.5 | - |
| Subtype (Blastoid/pleomorphic vs Classical/leukaemic non-nodal) | 1.38 (0.74-2.58), p=0.3 | - |
| TP53 mutation (Yes vs No) | 1.62 (0.74-3.55), p=0.2 | - |
| Stage  I-II  III  IV | Reference  1.91 (0.48-7.64), p=0.4  2.44 (0.76-7.86), p=0.1 | - |
| History of CNS disease (Yes vs No) | 4.53 (1.39-14.76), p=0.01 | 5.75 (1.29-25.69), p=0.02 |
| Bulk (>5cm) (Yes vs No) | 2.01 (1.18-3.42), p=0.01 | - |
| LDH (Elevated vs Normal) | 2.22 (1.28-3.83), p=0.004 | - |
| EN sites (≥3 vs <3) | 1.25 (0.54-2.93), p=0.6 | - |
| Prior lines (≥3 vs 2) | 1.21 (0.71-2.07), p=0.5 | - |
| POD24 (Yes vs No) | 1.62 (0.92-2.87), p=0.1 | 2.03 (1.09-3.77), p=0.03 |
| Primary refractory (Yes vs No) | 2.27 (1.06-4.83), p=0.03 | - |
| Ibrutinib refractory (Yes vs No) | 1.84 (1.06-3.20), p=0.03 | - |
| Previous ASCT (Yes vs No) | 0.56 (0.31-1.02), p=0.06 | - |
| Previous Allo-SCT (Yes vs No) | 0.72 (0.31-1.68), p=0.4 | - |
| **Pre-apheresis** |  |  |
| Prior bendamustine (Yes vs No) | 1.67 (0.98-2.83), p=0.06 | - |
| **Pre-LD** |  | - |
| ECOG PS  0  1  2 | Reference  1.22 (0.67-2.21), p=0.5  3.79 (1.56-9.17), p=0.003 | - |
| Bulk (>5cm) (Yes vs No) | 2.01 (1.12-3.60), p=0.02 | - |
| LDH (Elevated vs Normal) | 2.06 (1.17-3.62), p=0.01 | 2.20 (1.20-4.02), p=0.01 |
| Platelets (<75x10^9^/L vs ≥75x10^9^/L) | 3.02 (1.71-5.33), p<0.001 | 3.24 (1.77-5.94), p<0.001 |
| EN sites (≥3 vs <3) | 2.73 (1.37-5.47), p=0.005 | - |
| Bridging response (CR/PR vs SD/PD) | 1.14 (0.65-2.01), p=0.6 | - |

Variables in MVA were selected using forward stepwise selection (p-value for inclusion = 0.05) with the following variables considered: age, sex, ECOG PS pre-LD, sMIPI, HCT-CI, stage, CNS history, number of prior lines, POD24, ibrutinib refractory, prior ASCT, prior Allo-SCT, prior Bendamustine, bulk >5cm pre-LD, pre-LD LDH, pre-LD platelets, ≥3 EN sites pre-LD and bridging response.

Variables with >20% missing data (Ki67, subtype, TP53 mutation) were not included as they would reduce sample size too much for analysis. Pre-LD measurements were prioritised over submission measurements where both were available. Primary refractoriness was not included due to overlap with ibrutinib refractoriness.

**Table S6: Severe Infection by day 30 by BT modality and response**

| **BT modality** | **Severe infection within 30 days** | **No severe infection within 30 days** |
| --- | --- | --- |
| No BT/steroids only  (N=13) | 5/12 (42%) | 7/12 (58%) |
| Targeted (N=33) | 6/30 (20%) | 24/30 (80%) |
| RT +/- targeted  (N=19) | 4/12 (33%) | 8/12 (67%) |
| Low dose chemo  (N=3) | 0 | 2/2 (100%) |
| SD chemo +/-RT  (N=79) | 26/77 (36%) | 51/77 (66%) |
| **BT response** |  |  |
| CR  (N=19) | 7/16 (44%) | 9/16 (56%) |
| PR  (N=49) | 14/44 (32%) | 30/44 (68%) |
| SD  (N=30) | 5/30 (17%) | 25/30 (83%) |
| PD  (N=35) | 10/30 (33%) | 20/30 (66%) |

**Table S7 (a): Rates of ≥grade 3 Neutropenia and thrombocytopenia by BT modality**

| **BT modality** | **Thrombocytopenia** | | **Neutropenia** | |
| --- | --- | --- | --- | --- |
|  | **At M1** | **At M3** | **At M1** | **At M3** |
| No bridging/steroids only  (N=13) | 9/12 (75%) | 5/11 (45%) | 9/12 (75%) | 3/11 (27%) |
|  |  |  |  |  |
| Targeted  (N=33) | 10/31 (32%) | 4/26 (15%) | 15/31 (48%) | 5/26 (19%) |
|  |  |  |  |  |
| RT +/- targeted (N=19) | 10/17 (59%) | 3/16 (19%) | 9/17 (53%) | 3/16 (19%) |
|  |  |  |  |  |
| Low dose chemo  (N=3) | 1/2 (50%) | 0/2 (0%) | 1/2 (50%) | 1/2 (50%) |
|  |  |  |  |  |
| SD chemo +/- RT (N=79) | 57/73 (78%) | 28/57 (49%) | 54/74 (73%) | 19/57 (33%) |
|  |  |  |  |  |
| All patients (N=147) | 87/135 (64%) | 40/112 (36%) | 88/136 (65%) | 31/112 (28%) |

**Table S7 (b): Logistic regression of** $\boldsymbol{\geq}$**grade 3 thrombocytopenia and neutropenia by BT type**

|  | **Thrombocytopenia** | | **Neutropenia** | |
| --- | --- | --- | --- | --- |
|  | **Month 1** | **Month 3** | **Month 1** | **Month 3** |
| **Vs all other combined**  No BT/steroids only  Targeted  RT +/- targeted  Low dose chemo  SD chemo +/-RT | 1.73 (0.45-6.72), p=0.4  **0.17 (0.07-0.40), p<0.001**  0.76 (0.27-2.15), p=0.6  0.55 (0.03-8.94), p=0.7  **3.80 (1.80-8.01), <0.001** | 1.58 (0.45-5.52), p=0.5  **0.25 (0.08-0.80), p=0.02**  0.37 (0.10-1.38), p=0.1  N/A  **3.46 (1.52-7.89), p=0.003** | 1.71 (0.44-6.64), p=0.4  **0.41 (0.18-0.93), p=0.03**  0.57 (0.20-1.59), p=0.3  0.54 (0.03-8.83), p=0.7  **2.22 (1.09-4.55), p=0.03** | 0.98 (0.24-3.95), p>0.9  0.55 (0.19-1.62), p=0.3  0.56 (0.15-2.12), p=0.4  2.67 (0.16-44.00), p=0.5  1.79 (0.77-4.17), p=0.2 |

**Table S8 (a): Cumulative non-relapse mortality by BT modality**

| **NRM** | **No bridging/steroids**  **N=13** | **Targeted**  **N=33** | **RT +/- targeted**  **N=19** | **Low dose chemo**  **N=3** | **SD chemo +/-RT**  **N=79** |
| --- | --- | --- | --- | --- | --- |
| 6-month | 15% (4-49) | 0% | 0% | 33% (5-95) | 14% (8-24) |
| 12-month | 15% (4-49) | 0% | 7% (1-42) | 33% (5-95) | 21% (13-32) |
| 24-month | 33% (11-77) | 5% (1-33) | 7% (1-42) | N/A | 29% (19-44) |
|  |  |  |  |  |  |
| **SHR^1^ (95% CI), p-value:** | |  |  |  |  |
| vs None | - | 0.07 (0.01-0.51)  p=0.009 | 0.29 (0.06-1.31)  p=0.1 | 1.74 (0.15-19.9)  p=0.7 | 0.74 (0.28-1.99)  p=0.6 |
| vs Targeted | 15.04 (1.97-114.67)  p=0.009 | - | 4.07 (0.38-43.46)  p=0.2 | 14.09 (1.02-194.29)  p=0.05 | 9.06 (1.22-67.30)  p=0.03 |
| vs RT +/- targeted | 3.43 (0.76-15.42)  p=0.1 | 0.25 (0.02-2.62)  p=0.2 | - | 6.43 (0.42-98.87)  p=0.2 | 2.38 (0.63-8.99)  p=0.2 |
| vs Low dose chemo | 0.57 (0.05-6.56)  p=0.7 | 0.07 (0.01-0.98)  p=0.05 | 0.16 (0.01-2.39)  p=0.2 | - | 0.54 (0.05-5.69)  p=0.6 |
| vs SD chemo +/-RT | 1.34 (0.50-3.58)  p=0.6 | 0.11 (0.01-0.82)  p=0.03 | 0.42 (0.11-1.58)  p=0.2 | 1.84 (0.18-19.27)  p=0.6 | - |

^1^SHR: Sub distribution hazard ratio

**Table S8 (b): Early non-relapse mortality (within 90 days) by BT modality**

| **Infused patients^1^** | **No bridging/steroids**  **N=13** | **Targeted**  **N=31** | | **RT +/- targeted**  **N=17** | | **Low dose chemo**  **N=3** | | **SD chemo +/-RT**  **N=75** | |  |
| --- | --- | --- | --- | --- | --- | --- | --- | --- | --- | --- |
| Early NRM^2^ | 2 (15%) | 0 | | 0 | | 1 (33%) | | 10 (13%) | |  |
| No early NRM | 11 (85%) | 31 (100%) | | 17 (100%) | | 2 (67%) | | 65 (87%) | |  |
|  |  |  | |  | |  | |  | |  |
| **Fisher’s exact p-value:** | | |  | |  | |  | |  | |
| vs None | - | 0.08 | | 0.2 | | 0.5 | | 1 | |  |
| vs Targeted | 0.08 | - | | N/A | | 0.09 | | 0.03 | |  |
| vs RT +/- targeted | 0.2 | N/A | | - | | 0.2 | | 0.2 | |  |
| vs Low dose chemo | 0.5 | 0.09 | | 0.2 | | - | | 0.4 | |  |
| vs SD chemo +/-RT | 1 | 0.03 | | 0.2 | | 0.4 | | - | |  |

^1^Denominator excludes patients with <90 days follow-up post-infusion
^2^Early NRM: Early non-relapse mortality defined as death without relapse within 90 days of infusion

**Table S9: Univariate analysis of factors associated with early NRM post-infusion**

|  | **UVA OR (95% CI), p-value^1^** |
| --- | --- |
| **At submission** |  |
| Age (10-year increase) | 1.45 (0.63-3.35), p=0.4 |
| Sex (Male vs Female) | p=0.07^2^ |
| ECOG (1 vs 0) | 3.63 (0.76-17.26), p=0.1 |
| sMIPI  Low or Intermediate  High | Reference  5.41 (1.07-27.24), p=0.04 |
| HCT-CI  0  1 or 2  ≥3 | Reference  1.12 (0.30-4.22), p=0.9  0.57 (0.06-5.08), p=0.6 |
| Ki67 (≥30% vs <30%) | 1.38 (0.15-12.66), p=0.8 |
| Subtype (Blastoid/pleomorphic vs Classical/leukaemic non-nodal) | 2.92 (0.71-12.01), p=0.1 |
| TP53 mutation (Yes vs No) | 1.94 (0.25-14.97), p=0.5 |
| Stage  I-II  III  IV | Reference  0.94 (0.05-16.37), p>0.9  1.65 (0.20-13.83), p=0.6 |
| History of CNS disease (Yes vs No) | p=1^2^ |
| Bulk (>5cm) (Yes vs No) | 1.31 (0.40-4.28), p=0.7 |
| LDH (Elevated vs Normal) | 14.36 (1.81-113.85), p=0.01 |
| EN sites (≥3 vs <3) | 2.00 (0.39-10.31), p=0.4 |
| Prior lines (≥3 vs 2) | 0.50 (0.13-1.93), p=0.3 |
| POD24 (Yes vs No) | 0.35 (0.10-1.20), p=0.09 |
| Primary refractory (Yes vs No) | 0.86 (0.10-7.22), p=0.9 |
| Ibrutinib refractory (Yes vs No) | 1.45 (0.45-4.74), p=0.5 |
| Previous ASCT (Yes vs No) | 1.49 (0.47-4.70), p=0.5 |
| Previous Allo-SCT (Yes vs No) | p=0.2^2^ |
| **Pre-apheresis** |  |
| Prior bendamustine (Yes vs No) | 0.72 (0.21-2.47), p=0.6 |
| **Pre-LD** |  |
| ECOG PS  0  1  2 | Reference  2.13 (0.42-10.70), p=0.4  15.00 (2.25-100.20), p=0.01 |
| Bulk (>5cm) (Yes vs No) | 1.44 (0.35-5.83), p=0.6 |
| LDH (Elevated vs Normal) | 5.98 (1.25-28.55), p=0.03 |
| Platelets (<75x10^9^/L vs ≥75x10^9^/L) | 0.33 (0.10-1.10), p=0.07 |
| EN sites (≥3 vs <3) | 2.06 (0.39-10.77), p=0.4 |
| Standard chemo +/- RT (vs all other BT) | 3.13 (0.82-11.91), p=0.09 |
| Bridging response (CR/PR vs SD/PD) | 1.61 (0.43-6.01), p=0.5 |
| **Post-infusion** |  |
| ≥grade 3 neutropenia at M1 | p=0.03^2^ |
| ≥grade 3 thrombocytopenia at M1 | p=0.05^2^ |
| Severe/life-threatening infection within 30 days | 3.52 (1.04-11.88), p=0.04 |
| ≥grade 3 CRS | 2.20 (0.54-8.91), p=0.3 |
| ≥grade 3 ICANS | 6.46 (1.95-21.46), p=0.002 |

^1^Odds ratios, 95% confidence intervals and p-values calculated using logistic regression unless stated otherwise. ^2^P-value from Fisher’s exact test as no early NRM events in the following groups: female, history of CNS, previous allo-SCT, without G3+ neutropenia at month 1, without G3+ thrombocytopenia at month 1

**Table S10: Cause of early and late non-relapse mortality (NRM) by BT modality**

| **Timing of NRM** | **Day of Death post infusion** | **BT modality** | **Cause of death** | **Neutrophil recovery at time of death (>0.5)** |
| --- | --- | --- | --- | --- |
| **Early (<90 days)** | | | | |
| Early | 6 | SD chemo | Suspected grade 5 CRS | No |
| Early | 9 | Low dose chemo | Sepsis in context of ICANS | No |
| Early | 14 | SD chemo | Sepsis in context of ICANS | No |
| Early | 16 | SD chemo | ICANS/HLH | No |
| Early | 28 | SD chemo | Ischaemic heart disease | No |
| Early | 29 | SD chemo | Gram negative sepsis | No |
| Early | 32 | No bridging | Sepsis (presumed bacterial) | No |
| Early | 34 | SD chemo | Gram negative sepsis/bowel perforation | No |
| Early | 36 | SD chemo | Fungal pneumonia in context of ICANS | Yes |
| Early | 38 | SD chemo | Gram negative sepsis | No |
| Early | 57 | SD chemo + RT | Gram positive sepsis | Yes |
| Early | 56 | SD chemo | Gram negative Sepsis | No |
| Early | 82 | Steroids | Cytopenia, declined further care | No |
| **Late (≥90 days)** | | | | |
| Late | 105 | SD chemo | Hospital acquired pneumonia | NA |
| Late | 224 | SD chemo | Recurrent infections | NA |
| Late | 231 | SD chemo | SARS-CoV 2 | NA |
| Late | 258 | SD chemo+ RT | SARS-CoV2 | NA |
| Late | 306 | SD chemo | Pulmonary fibrosis | Yes |
| Late | 307 | SD chemo | Increasing frailty + transfusion requirements | NA |
| Late | 392 | No bridging | Acute myeloid leukaemia | NA |
| Late | 400 | SD chemo | Infection | NA |
| Late | 479 | SD chemo +RT | Progressive physical decline with cognitive dysfunction | Yes |
| Late | 522 | SD chemo | Acute myeloid leukaemia | NA |
| Late | 638 | SD chemo | SARS-CoV2 | NA |
| Late | 783 | SD chemo | Klebsiella pneumonia | NA |
| Late | 965 | RT | Recurrent chest infections | NA |
| Late | 974 | No bridging | SARS-CoV2 | NA |

**Table S11: Impact of BT response on variables of disease burden pre-LD**

|  | **No response to BT** | **Any response to BT** | **CR to BT** |
| --- | --- | --- | --- |
| LDH >ULN pre-LD | 32/65 (49%) | 32/59 (54%) | 11/18 (61%) |
| Bulk>5cm pre-LD | 18/63 (29%) | 10/65 (15%) | 0/19 (0%) |
| 3+ EN sites pre-LD | 10/63 (16%) | 4/62 (6%) | 0/18 (0%) |
| ECOG ≥1 pre-LD | 50/73 (68%) | 46/70 (66%) | 11/19 (58%) |

**Supplementary Figures:**

**Supp Figure 1**: Time from harvest to CAR T infusion by modality of bridging therapy

(Kruskal-Wallis test p-value= 0.7)


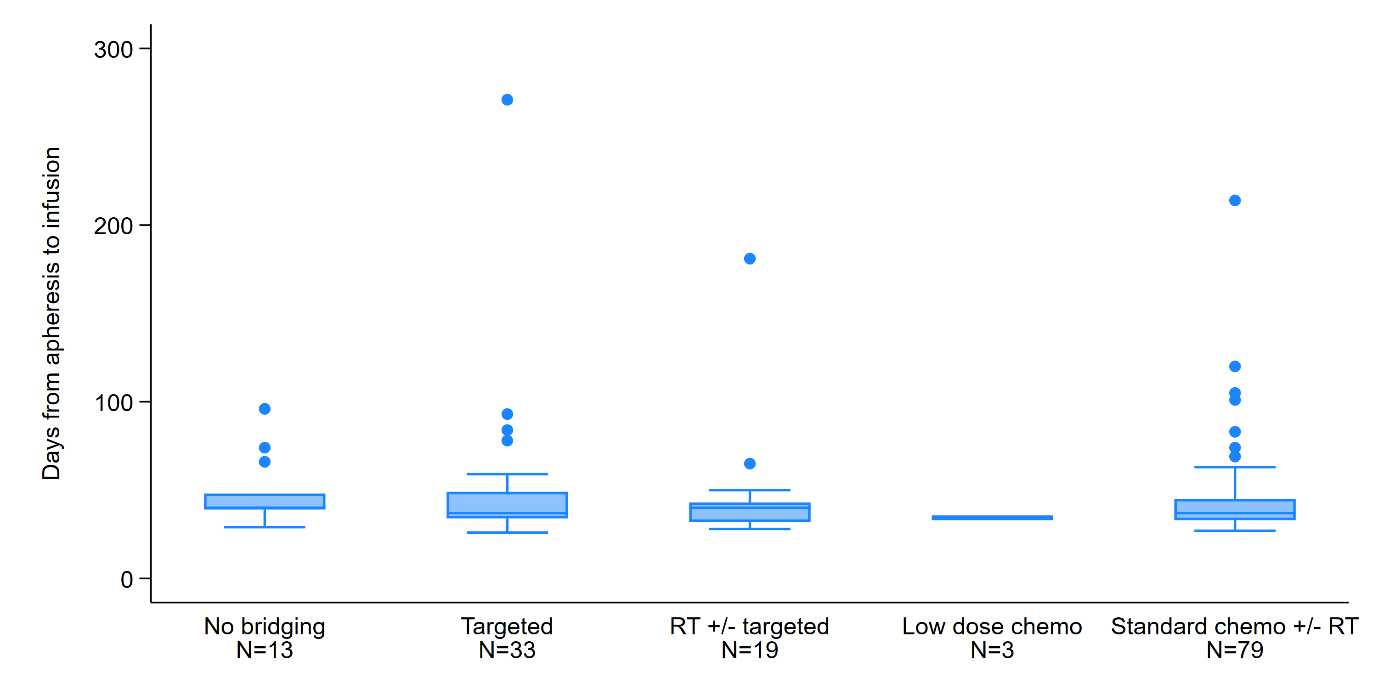


**Supp Figure 2 (a, b):** (a) PFS by targeted therapy vs standard chemo +/-RT (b) OS by targeted therapy vs standard chemo +/-RT

PFS HR=1.78, 95% CI 0.85-3.71, p=0.1; OS HR=1.86, 95% CI 0.89-3.89, p=0.1

**(a)**


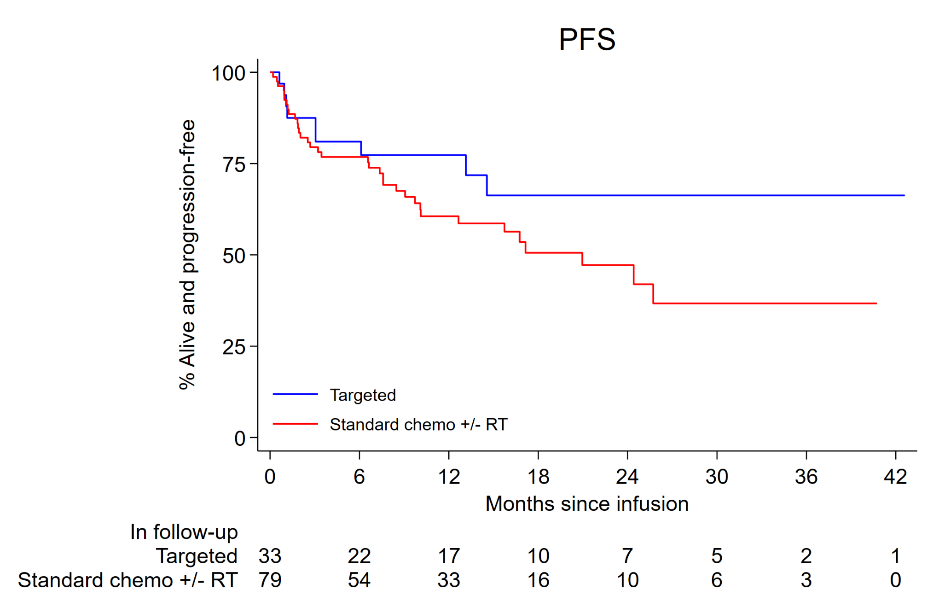


**(b)**


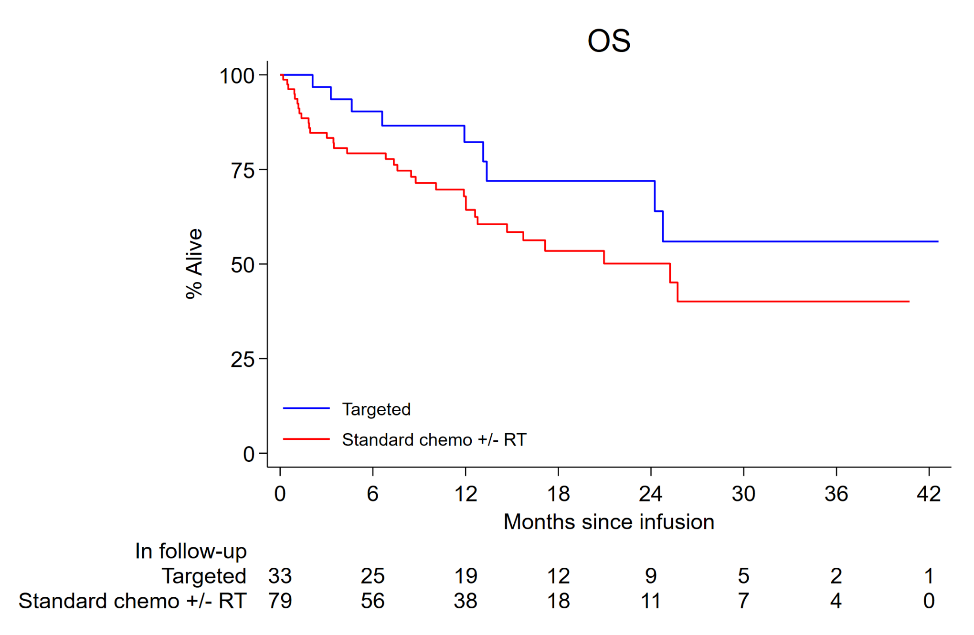


**Supp Figure 3 (a, b):** Highest grade CRS (a) and ICANS (b) by response to BT

**(a)**


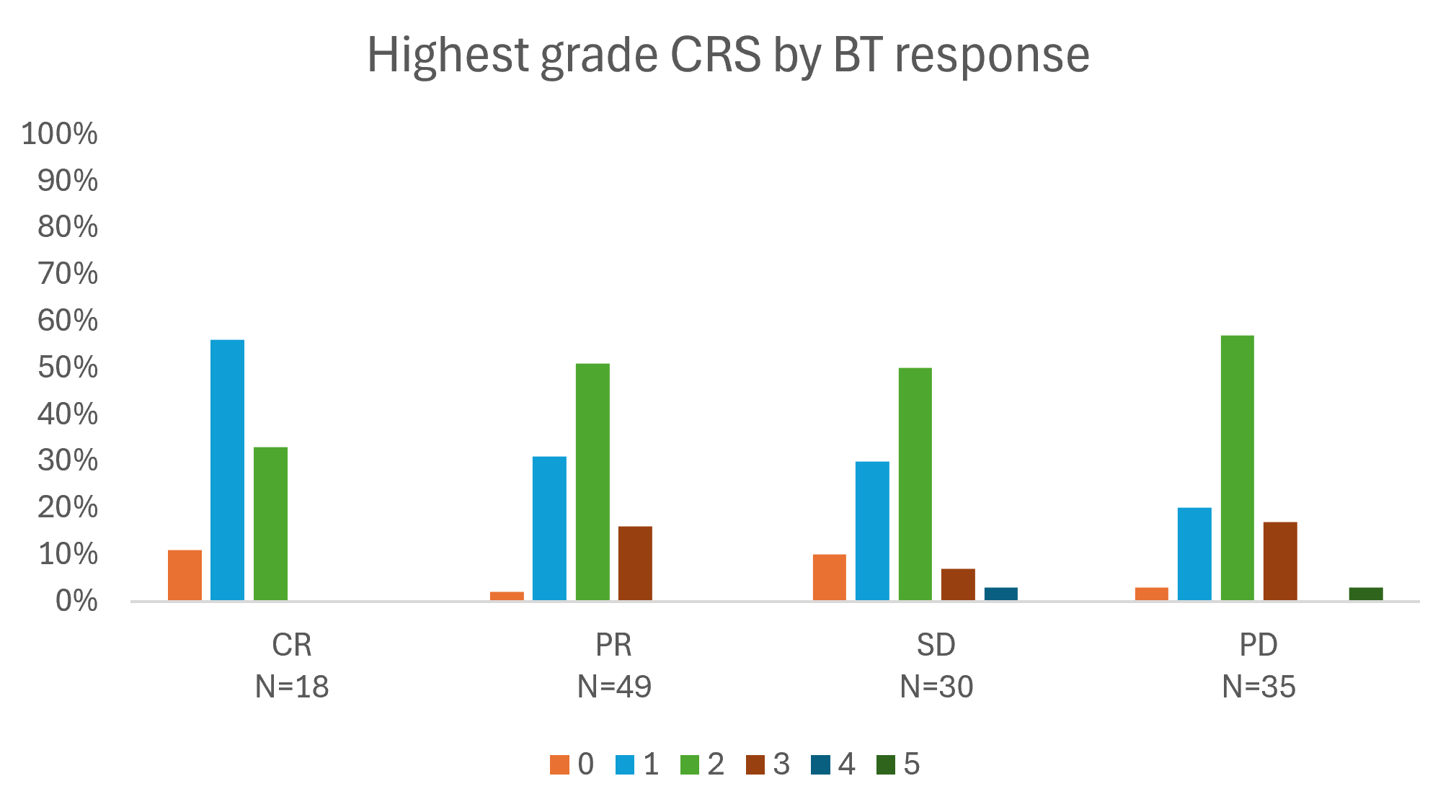


**(b)**


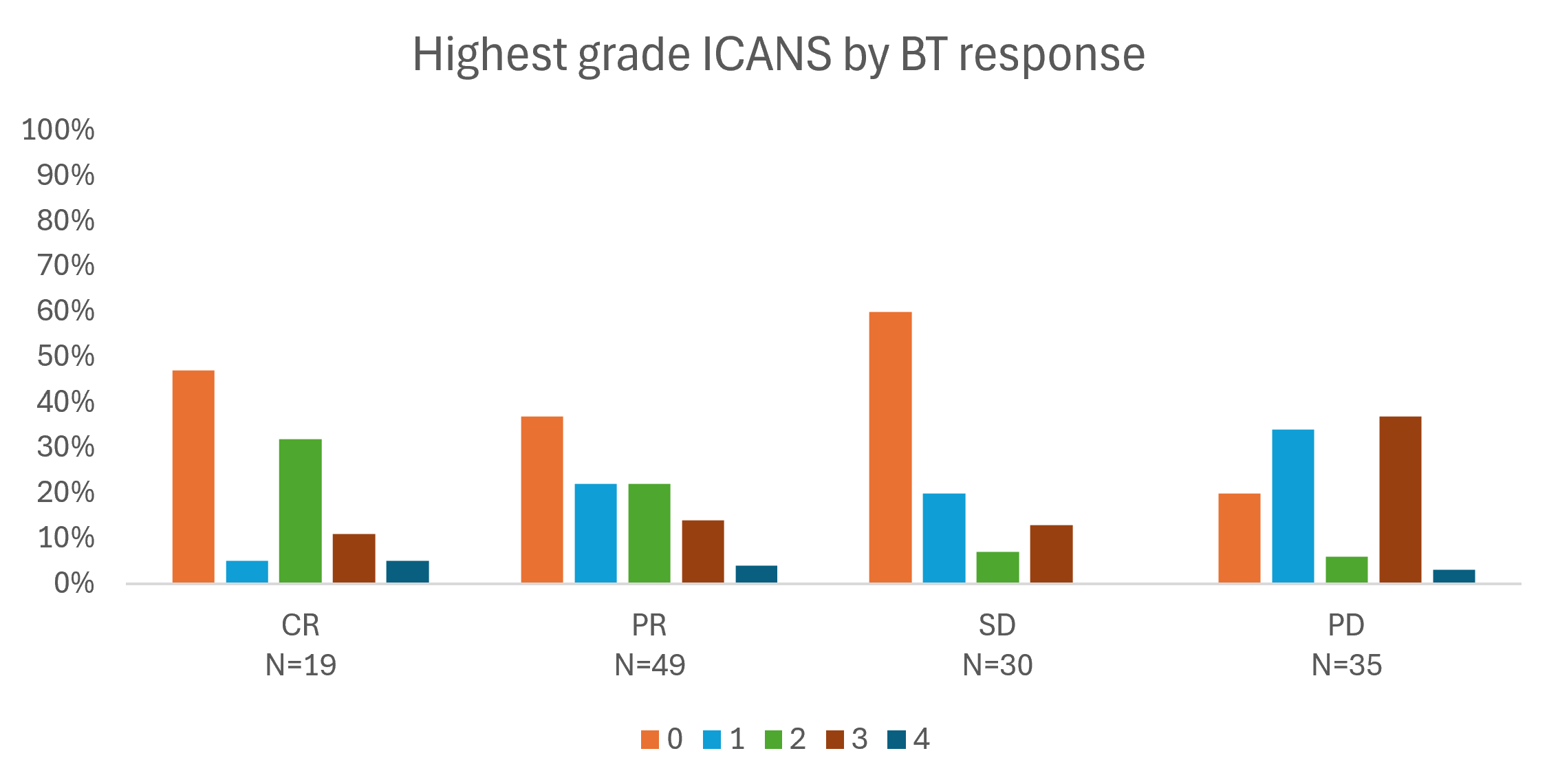


**Supp Figure 4(a):** Non-relapse mortality by type of BT


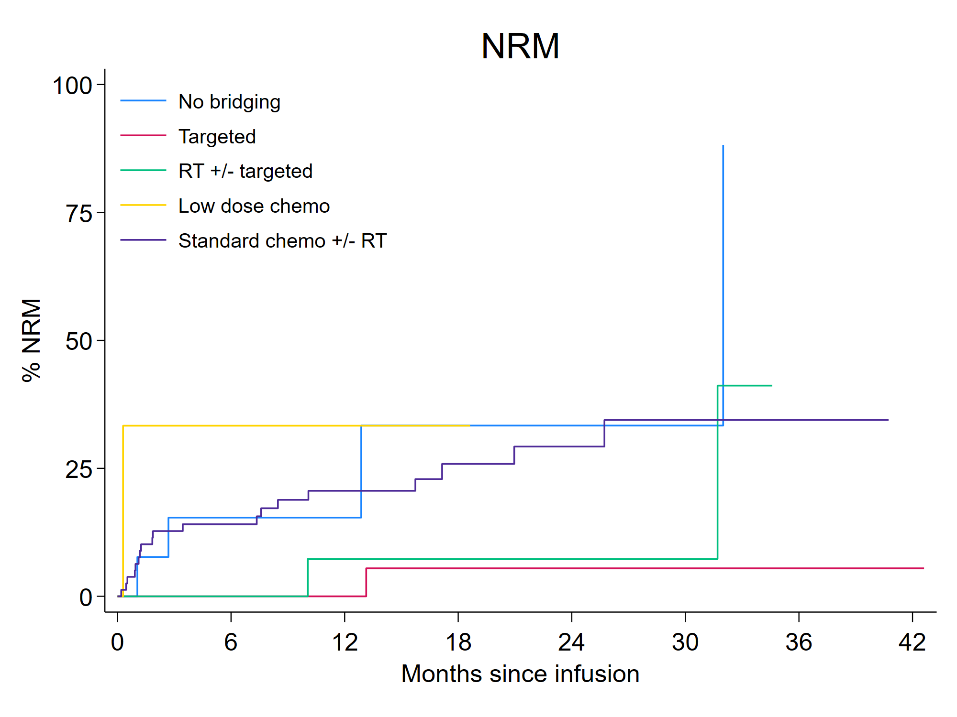


**Supp Figure 4(b):** Non-relapse mortality by R-BAC vs other BT

UVA: SHR = 3.20 (1.39-7.34), p=0.01; MVA (adjusted for ECOG PS and LDH at submission): SHR = 2.17 (0.83-5.63), p=0.1


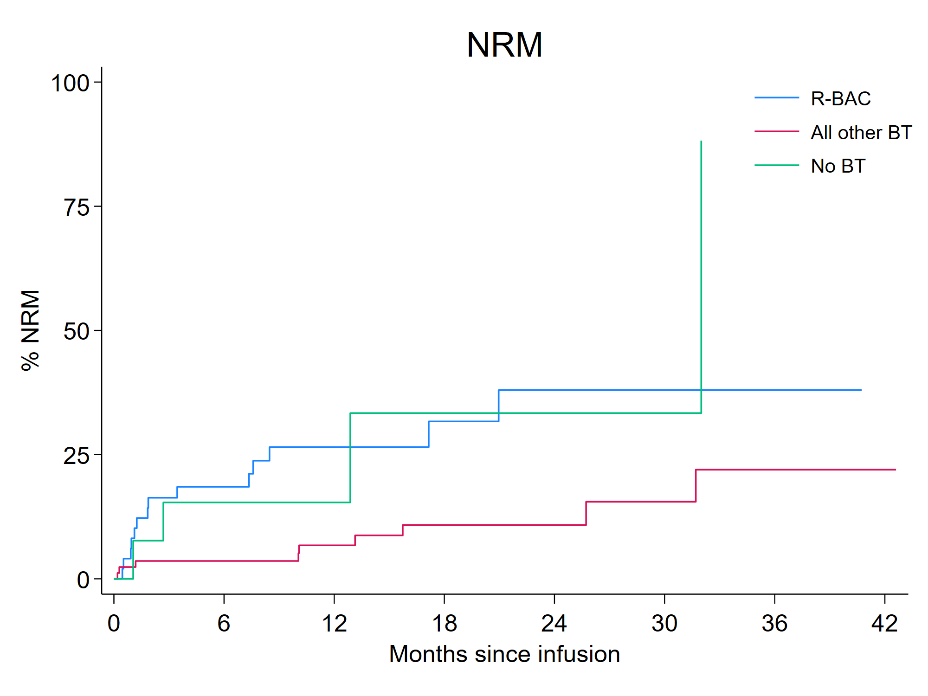


**Supp Figure 4 (c):** Early NRM (within 90 days) by choice of BT


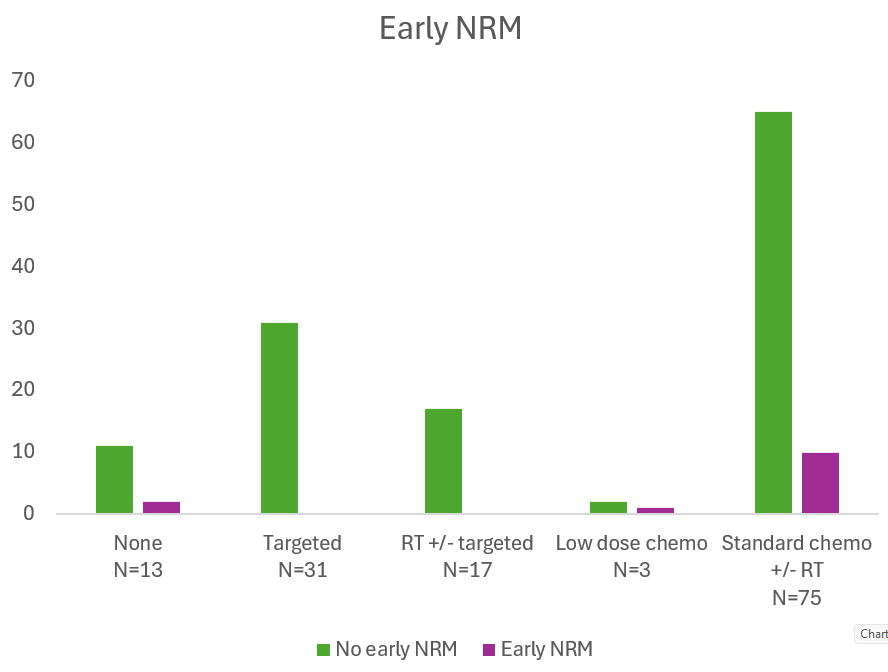


**Supp Figure 5:** Non-relapse mortality by response to BT


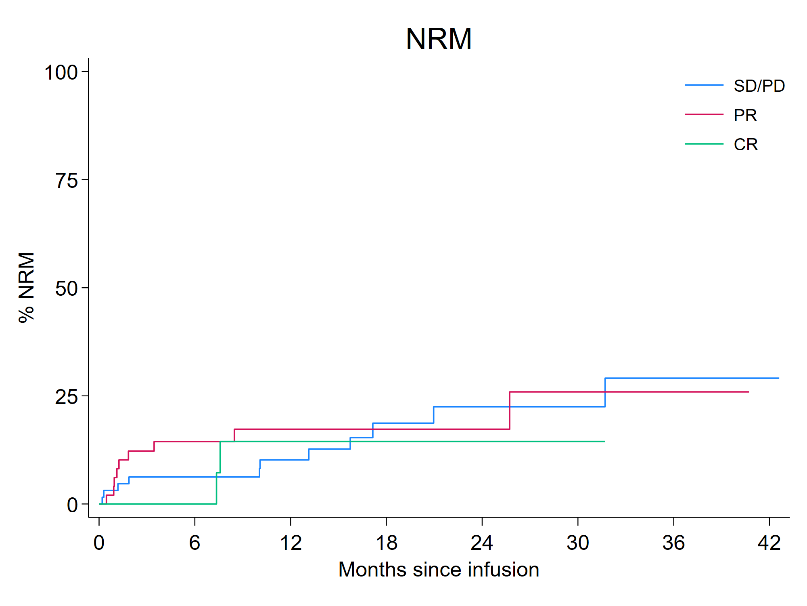

Supplement: Supplementary file 1 — Data S1. [file BJH-208-1347-s001.docx]
